# Complete Genome Sequence of *Helicobacter pylori* Strain 7C Isolated from a Mexican Patient with Chronic Gastritis

**DOI:** 10.1128/genomeA.01503-15

**Published:** 2016-01-07

**Authors:** Eduardo Mucito-Varela, Gonzalo Castillo-Rojas, Miguel A. Cevallos, Luis Lozano, Enrique Merino, Gamaliel López-Leal, Yolanda López-Vidal

**Affiliations:** a Departamento de Microbiología y Parasitología, Facultad de Medicina, Programa de Inmunología Molecular Microbiana, Universidad Nacional Autónoma de México (UNAM), Ciudad de México, Distrito Federal, Mexico; b Centro de Ciencias Genómicas, Programa de Genómica Evolutiva, Universidad Nacional Autónoma de México (UNAM), Cuernavaca, Morelos, Mexico; c Instituto de Biotecnología, Universidad Nacional Autónoma de México (UNAM), Cuernavaca, Morelos, Mexico; d Facultad de Medicina, División de Investigación, Universidad Nacional Autónoma de México (UNAM), Ciudad de México, Distrito Federal, Mexico

## Abstract

*Helicobacter pylori*-induced gastritis is a risk factor for developing gastric pathologies. Here, we report the complete genome sequence of a multidrug-resistant *H. pylori* strain isolated from a chronic gastritis patient in Mexico City, Mexico. Nonvirulent VacA and *cag*-pathogenicity island (PAI) genotypes were found, but the presence of a potential mobilizable plasmid carrying an IS*605* element is of outstanding interest.

## GENOME ANNOUNCEMENT

*Helicobacter pylori* infection produces chronic gastritis and increases the risk of peptic ulcer, gastric cancer, and mucosa-associated lymphoid tissue (MALT) lymphoma due to the bacterial interaction between its virulence factors and the cellular components of the host (1, 2). Whether the genome structure of the *H. pylori* strains isolated in Mexico is similar to that of the strains isolated worldwide or not remains unknown, as only one genome of *H. pylori* isolated in Mexico has been sequenced (3).

Here, we present the complete genome sequence of *H. pylori* strain 7C, which was isolated from a patient with chronic gastritis in Mexico City, Mexico. It is a multidrug-resistant strain, and, in a previous study, it was found to increase the apoptosis of the AGS human gastric epithelial cell line associated with the overexpression of cytosolic and membrane proteins of the bacterium (4). The genome was *de novo* assembled using a combination of PacBio RS, Roche 454, and Illumina MiSeq reads, with a total average coverage of 121×. Genome annotation was performed using the NCBI Prokaryotic Genome Annotation Pipeline (http://www.ncbi.nlm.nih.gov/genome/annotation_prok/). The *H. pylori* 7C chromosome is 1,624,441 bp in length, with a G+C content of 39%. It contains 1,553 genes, 83 pseudogenes, 36 tRNAs, 6 rRNAs, and 1 noncoding RNA (ncRNA). Additionally, a sequence analysis of this strain showed the absence of the *cag* pathogenicity island and s2i2m2 VacA genotype. Moreover, *H. pylori* 7C contains a 6,835-bp plasmid similar to other *H. pylori* plasmids previously reported in the NCBI genome database. This plasmid carries genes encoding 11 proteins that include a transposase of the IS*605* insertion element, a replicase, mobilization gene (mobABD), and four hypothetical proteins. This genome and other sequenced genomes will be used for comparative genomics analyses.

### Nucleotide sequence accession numbers.

This whole-genome shotgun project has been deposited in GenBank under the accession numbers CP012905 and CP012906.
